# Diagnosis of a Rare *Rickettsia felis* Infection Complicated with Unusual Pericardial Effusion and Cardiac Tamponade Using an mNGS Test

**DOI:** 10.1155/2024/8877876

**Published:** 2024-08-13

**Authors:** Tien-Lung Po, Chien-Hsien Huang, Chia-Hsun Lin, Huei-Fong Hung

**Affiliations:** ^1^ Division of Cardiology Department of Internal Medicine Shin Kong Wu Ho-Su Memorial Hospital, Taipei, Taiwan; ^2^ Department of Internal Medicine Shin Kong Wu Ho-Su Memorial Hospital, Taipei, Taiwan; ^3^ Division of Infectious Disease Department of Internal Medicine Shin Kong Wu Ho-Su Memorial Hospital, Taipei, Taiwan; ^4^ College of Medicine Fu Jen Catholic University, Hsinchuang, Taipei, Taiwan; ^5^ Division of Cardiac Surgery Department of Surgery Shin Kong Wu Ho-Su Memorial Hospital, Taipei, Taiwan; ^6^ Department of Surgery Shin Kong Wu Ho-Su Memorial Hospital, Taipei, Taiwan

## Abstract

The occurrence of sporadic rickettsial infections has been consistently undervalued and overlooked, primarily owing to a limited emphasis on routine examinations for rickettsioses in clinical practice. At present, the immunofluorescence assay is the prevailing diagnostic method for suspected rickettsioses that enables the detection of specific antibodies against rickettsia in human serum. Herein, we present an exceptional instance of rickettsial infection that was characterized by a rare manifestation of extensive pericardial effusion leading to dyspnea and cardiac tamponade. A diagnosis of chronic fibrosing pericarditis was established based on pericardium tissue obtained through pericardiotomy, and a conclusive metagenomic next-generation sequencing test confirmed the presence of *Rickettsia felis* infection. The cat flea, scientifically known as *Ctenocephalides felis*, is the predominant carrier of *R. felis*. An escalating incidence of human *R. felis* infections has raised concerns, particularly in light of the burgeoning population of domesticated animals in many contemporary societies.

## 1. Introduction

Among the expansive array of arthropods, including fleas, ticks, mites, and lice, the cat flea (*Ctenocephalides felis*) is widely acknowledged as the principal reservoir of *Rickettsia felis* bacteria [1]. A recent report highlighted the emergence of mosquitoes as carriers of *R. felis* in the United States [2].

The clinical manifestations of human *R. felis* infection, commonly referred to as flea-borne spotted fever or cat flea typhus, are similar to those of other rickettsial diseases. These similarities manifest as symptoms including fever, rash, myalgia, and headache. Furthermore, in more severe cases, individuals may exhibit additional manifestations such as hepatomegaly, myocarditis, meningoencephalitis, and acute respiratory distress syndrome [3].

Although it was initially recognized as a pathogenic agent in humans in the United States in 1994 [4], *R. felis* (an intracellular gram-negative bacterium) continues to be underestimated, despite advancements in serological and molecular diagnostic techniques such as immunofluorescence assay (IFA) and polymerase chain reaction (PCR). This lack of recognition can be attributed to the limited emphasis placed on the routine examination of rickettsioses in clinical practice. Consequently, it is possible that a significant proportion of human cases are not accurately diagnosed.

In this report, we present a unique case of *R. felis* infection that was characterized by an uncommon clinical manifestation of dyspnea and cardiac tamponade caused by extensive pericardial effusion. The diagnosis was validated through an examination of the collected pericardial fluid using a novel metagenomic next-generation sequencing (mNGS) test, which provided conclusive results.

## 2. Case Presentation

A 79-year-old female patient was admitted to the intensive care unit with dyspnea lasting one week and cardiac tamponade, which was determined through echocardiography to be caused by substantial pericardial effusion of unclear origin. Her medical history included the following: (1) a history of breast cancer with a left mastectomy in 2008 and a partial right mastectomy in 2015; (2) prior instances of ventricular fibrillation and long QT syndrome, which prompted the implantation of an implantable cardioverter-defibrillator (ICD) in 2013; (3) paroxysmal atrial fibrillation under direct oral anticoagulants (DOAC) since 2013; (4) type 2 diabetes mellitus; and (5) hyperlipidemia.

**Figure 1:**
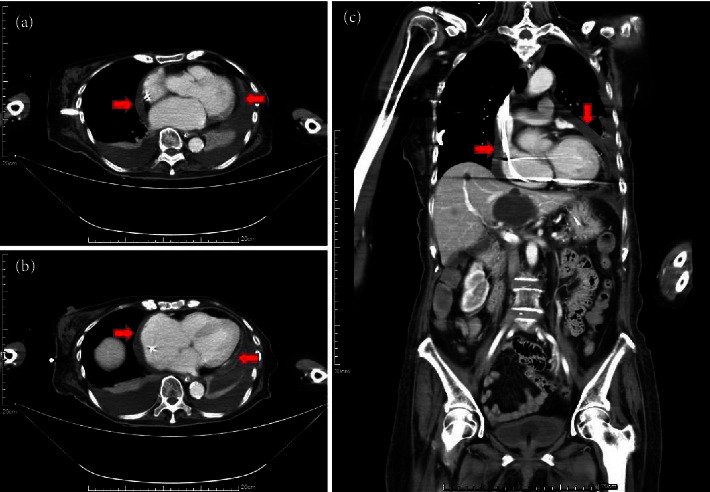
Transverse plane (a, b) and coronal plane (c) computed tomography (CT): observed pericardial effusion (red arrows) with relative hyperdensity of the collections and suspected cardiac tamponade.

Computed tomography revealed bilateral pleural effusion and suggested the presence of cardiac tamponade caused by a substantial pericardial effusion (Figure 1). Initially, a pericardiocentesis procedure extracted 1030 cc of dark red drainage. The patient was additionally administered 4.5 g of tazocin (sodium piperacillin 2.0 g + sodium tazobactam 0.25 g) intravenously every 8 hours for 14 days, as part of a routine antibiotic treatment for suspected lung infection.

In response to the recurrent accumulation of pericardial and pleural effusions, a surgical pericardial-pleural window and pleurodesis were performed. To test for potential *tuberculosis*, the collected bloody pericardial fluid was subjected to routine analysis, cytology, and culture. Additionally, two pieces of pericardial tissue (measuring 3 × 4 cm and 2 × 6 cm) were excised anterior to the phrenic nerve for pathological examination.

**Figure 2:**
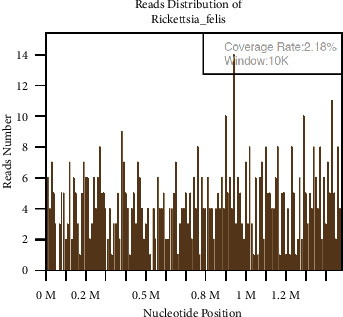
Results of mNGS analysis on the patient's pericardial fluid. The sequenced gene fragment regions were widely distributed across different locations on the *Rickettsia felis* genome. This indicated that the detected reads did not originate from single-fragmented DNA pieces through repeated sequencing but rather came from the entire genome, thus proving the presence of *Rickettsia felis* in the sample.

Conclusive pathological findings from the pericardial fluid and tissue demonstrated an absence of malignant cells. A subsequent mNGS test was performed to examine the pericardial fluid and serum blood samples for the presence of other infectious pathogens, owing to negative results obtained on a *tuberculosis* test. Nucleic acids were extracted from the collected samples using a TIANamp Micro DNA Kit (Tiangen Biotech Co., Ltd, Beijing, China) and then used for library construction with a MGIEasy Cell-free DNA Library Prep Kit (MGI Tech Co., Ltd, Shenzhen, China) and high-throughput sequencing on the MGISEQ-200 platform (MGI Tech Co., Ltd, Shenzhen, China). High-quality sequencing data were generated by removing short (<35 bp), low-quality, and low-complexity reads. Human reads were removed by mapping to the human reference genome hg38 (GRCh38, December 2017) using the Burrows–Wheeler Aligner. The remaining data were compared to the Microbial Genome Database (https://ftp.ncbi.nlm.nih.gov/genomes/) using the Burrows–Wheeler Alignment tool v0.7.10-r789. Our mNGS results indicated the presence of *Rickettsia felis* (275 reads) that accounted for 2.18% of nucleotide sequence coverage and showed 62.29% relative abundance in the pericardial fluid (Table 1, Figure 2) but was not detected in the blood. This prompted the addition of oral doxycycline 100 mg twice daily to the treatment regimen for 10 days, in accordance with relevant treatment guidelines [5]. A thorough investigation of the patient's history, as well as that of her family, revealed that the potential pathogen originated from a domesticated cat that lived in the patient's household.

The patient recovered from the illness, showing regression of the lung infection on chest radiography, and a decrease in pleural and pericardial effusions. The patient was discharged after completing the doxycycline treatment.

## 3. Discussion

Rickettsial infections are categorized into four groups, based on phenotypic and genetic characteristics. These include (1) the ancestral group (AG); (2) the typhus group (TG)—of which *Rickettsia typhi* is the most common member; (3) the spotted fever group (SFG)—which includes *Rickettsia rickettsia*; and (4) the transitional group (TRG)—containing *Rickettsia felis* [6].

IFA has become the standard and most commonly used method to detect specific antibodies against *R. felis* in the serum. However, a notable limitation arises because of cross-reactivity among various Rickettsiae species. Higher concentrations of antibodies against *R. felis* may potentially aid in distinguishing *R. felis* infections from other rickettsial diseases [7]. An additional diagnostic technique used to detect *R. felis* infection involves the use of PCR to amplify specific gene fragments of *R. felis*—including *gltA*, *ompA*, *ompB*, and *17-kDa* antigen [6–8]. In contrast to conventional pathogen detection techniques, novel mNGS analysis yields fast and precise pathogen detection and identification [9–11].

*R. felis* infection is a global health concern in the human population, exhibiting a correlation with febrile ailments that can induce severe or even lethal complications. Its presenting symptoms resemble those observed in other rickettsial diseases—ranging from mild fever, skin rash, cutaneous eschar at the bite site, myalgia, and headache; to less frequent yet more intricate manifestations involving the visceral organs, neurological complications, and the heart [8].

Although relatively rare, a range of neurological manifestations associated with *R. felis* infection—including hearing loss, photophobia, meningitis, meningoencephalitis, and symptoms resembling polyneuropathy—were comprehensively summarized in a literature review conducted by Zeng et al., spanning the period between 2000 and 2020 [12].

While myocarditis and pericarditis have been extensively documented as the primary cardiac complications associated with various rickettsial infections–including *Rickettsia rickettsii*, *Rickettsia conorii*, *Rickettsia africae*, *Rickettsia japonica*, and *Rickettsia tsutsugamushi* [13–15]—there remains a dearth of case reports that have specifically documented cardiac complications related to *R. felis* infection. Pericardial effusion has been reported to be a manifestation of *Rickettsia tsutsugamushi* and *Rickettsia typhi* [16–18]. Additionally, a previous case study reported pericarditis and pericardial effusion associated with *Bartonella quintana* [19]. However, to date, no published reports have described pericarditis or pericardial effusion associated with *R. felis* infection.

To the best of our knowledge, this is the first case report to document *R. felis* infection accompanied by pericarditis and pericardial effusion substantiated by direct pathological evidence obtained from the pericardial fluid, which was confirmed through mNGS.

Misdiagnosis of this type of infection is frequent, owing to inadequate awareness of it and the limited availability of specific laboratory testing required to confirm *R. felis* involvement. A recent review hypothesized that ∼33% of cat fleas collected from companion dogs and cats in Taiwan are infected with *R. felis* (although the available data were limited) [6].

Nevertheless, Lai et al. emphasized the long-standing underappreciation and neglect of spotted fever group rickettsioses in southern Taiwan, particularly focusing on species that are closely associated with *R. felis* [20]. Likewise, Yang et al. conducted a retrospective seroepidemiological analysis in Taiwan involving 122 patients who were suspected of having rickettsioses but tested negative for scrub typhus, murine typhus, or *Q* fever [21]. This study revealed a seropositivity rate of 19%, indicating exposure to rickettsia. Among these cases, eight individuals had antibodies that were responsive to *R. felis*—of whom four showed evidence of ongoing *R. felis* infection and one, whose doxycycline treatment was discontinued because negative results for scrub typhus, *Q* fever, and murine typhus experienced a fatal outcome.

## 4. Conclusion

In contrast to other rickettsial diseases in Taiwan, which are primarily transmitted by arthropods in natural habitats, the emergence of *Rickettsia felis* infection is thought to be associated with the expanding population of companion animals in contemporary society [6, 22, 23]. Therefore, it is imperative to engage in proactive surveillance of patients with unidentified causes of pericardial effusion. Furthermore, beyond conventional antibody detection using IFA, mNGS provides a new method for surveying rare infectious pathogens.

## Figures and Tables

**Table 1:** Results of mNGS on our patient's pericardial fluid.

Genus				Species			
Type	Pathogen	Reads	Genus relative abundance (%)^*∗*^	Pathogen	Reads	Coverage (%)	Species relative abundance (%)^*∗∗*^
G-bacteria	*Rickettsia*	910	93.15	*Rickettsia felis*	275	2.18	62.29

^*∗*^Relative abundance at the genus level refers to the proportion of detected microorganisms within the genus in the entire sample. ^*∗∗*^Relative abundance at the species level refers to the proportion of detected microorganisms within the species in the entire sample.

## Data Availability

All relevant data supporting the conclusions of this article are included within the article.

## Abbreviations

AG: Ancestral group
DOAC: Direct oral anticoagulants
ICD: Implantable cardioverter-defibrillator
IFA: Immunofluorescence assay
mNGS: Metagenomic Next-Generation Sequencing
PCR: Polymerase chain reaction
SFG: Spotted fever group
TG: Typhus group
TRG: Transitional group.
